# The prevalence and severity of oral impacts on daily performances in Thai primary school children

**DOI:** 10.1186/1477-7525-2-57

**Published:** 2004-10-12

**Authors:** Sudaduang Gherunpong, Georgios Tsakos, Aubrey Sheiham

**Affiliations:** 1 Department of Epidemiology and Public Health, University College London, 1-19 Torrington Place, London WC1E 6BT, United Kingdom

**Keywords:** oral impacts, quality of life, children

## Abstract

**Background:**

Traditional methods of measuring oral health mainly use clinical dental indices and have been complemented by oral health related quality of life (OHRQoL) measures. Most OHRQoL studies have been on adults and elderly populations. There are no systematic OHRQoL studies of a population-based sample of children. The objective of this study was to assess the prevalence, characteristics and severity of oral impacts in primary school children.

**Methods:**

Cross-sectional study of all 1126 children aged 11–12 years in a municipal area of Suphanburi province, Thailand. An OHRQoL measure, Child-Oral Impacts on Daily Performances index (Child-OIDP) was used to assess oral impacts. Children were also clinically examined and completed a self-administered questionnaire about demographic information and oral behaviours.

**Results:**

89.8% of children had one or more oral impacts. The median impact score was 7.6 and mean score was 8.8. Nearly half (47.0%) of the children with impacts had impacts at very little or little levels of intensity. Most (84.8%) of those with impacts had 1–4 daily performances affected (out of 8 performances). Eating was the most common performance affected (72.9%). The severity of impacts was high for eating and smiling and low for study and social contact performances. The main clinical causes of impacts were sensitive tooth (27.9%), oral ulcers (25.8%), toothache (25.1%) and an exfoliating primary tooth (23.4%).

**Conclusions:**

The study reveals that oral health impacts on quality of life in Thai primary school children. Oral impacts were prevalent, but not severe. The impacts mainly related to difficulty eating and smiling. Toothache, oral ulcers and natural processes contributed largely to the incidence of oral impacts.

## Background

Contemporary concepts of health suggest that dental health should be defined in physical, psychological and social well-being terms in relation to dental status [1,2]. That is why Cohen and Jago considered that the greatest contribution of dentistry is to the improvement of quality of life because most oral diseases and their consequences interfere with, or have impacts on, daily life performances [3]. Therefore, disruptions in normal physical, psychological and social functioning are important considerations in assessing oral health. Despite these suggestions, traditional methods of measuring oral health use mainly clinical dental indices and focus on the absence or presence of oral diseases. They do not inform us about the oral well-being of people in terms of feelings about their mouths, or, for example, their ability to chew and enjoy their food. The inadequacy of the normative approach in measuring oral health has been recognised and lead to the development of measures of oral health-related quality of life (OHRQoL) [4].

A number of socio-dental or OHRQoL measures have been developed and used for assessing oral well-being and to describe oral impacts on people's quality of life [5]. Generally, they measure the extent to which oral conditions disrupt normal social role functioning and lead to major changes in behaviours, such as changes in ability to work or attend school, or undertake parental or household duties [6,7]. In addition to describing oral impacts on quality of life, some OHRQoL measures were designed specially to assist dental service planning by incorporating them with traditional normative measures in the process of dental needs assessment [8,9].

Most studies using OHRQoL to assess oral impacts of the mouth and teeth have been on adults and elderly populations. Few studies have been done on children possibly because no OHRQoL measures designed for use with children existed until recently. A single measure, dental pain, has been used on children in Malaysia [10] and in South Africa [11]. They found a high prevalence of pain that affected daily living. Similarly, a study in New Zealand found that most school children complained of at least one dental symptom [12]. To date, there are no systematic OHRQoL studies of a large population-based sample of children. In particular, the OHRQoL of primary school children, who are frequently the main target group for dental health services, has not been assessed. Therefore the objective of this study was to use an OHRQoL measure, the Child-OIDP, to assess the prevalence, characteristics and severity of oral impacts in primary school children.

## Methods

A cross-sectional survey was carried out in a municipal area of Muang district, Suphanburi province, Thailand. The sample was all 1,126 students aged 11–12 years, in the final year class of all primary schools (grade 6) in the area.

Data were collected through: a) an interview for oral impacts using the Child-OIDP [9], by one interviewer b) a self-administered questionnaire for demographic information such as age, sex and occupation of the father and mother, or male and female guardians [13] and oral health behaviours and c) an oral examination by four calibrated community dentists, mainly based on the WHO guidelines [14]. Orthodontic normative treatment needs were assessed by the Index of Orthodontic Treatment Need (IOTN) [15]. Oral hygiene was also assessed using the Simplified-Oral Hygiene Index (OHI-S) [16]. All documents were translated from English to Thai and the validity was checked by a back-translation method, involving blind re-translation into English. The validity of the translation was verified by experts in the use of questionnaires in both languages. This was also checked after wording modifications, in order to ensure the conceptual and functional equivalences of the questionnaires. A pilot study was carried out to validate all questionnaires before using them in the main data collection. The psychometric properties of the Child-OIDP in terms of face, content and concurrent validity as well as internal and test-retest reliability were excellent. The index was also practical to use with this age group. Full description of the validation process of the Child-OIDP can be found elsewhere [9]. For the main data collection, test-retest reliability of data was tested by ten percent random duplication. Weighted kappa score for the Child-OIDP was 0.91, kappa scores of self-administered questionnaires were 0.7–1.0, and those of intra- and inter-examiner for oral examinations were 0.7–1.0 and 0.6–1.0 respectively indicating good to excellent agreement. The SPSS and Stata programmes were used for statistical analysis.

The protocol of the study was approved by the Ethical Committee of the Ministry of Public Health of Thailand. Primary education and local health authorities as well as all primary schools in the study areas gave permission. Positive consent forms and letters informing parents were sent to parents.

### Measuring oral impacts and calculating their severity

Two comprehensive OHRQoL measures specifically for use with pre-adolescent children have recently been developed; the Child Perceptions Questionnaire (CPQ11-14) [17] and the Child Oral Impacts on Daily Performances (Child-OIDP) [9]. Both were validated on a cross-sectional study using a proxy, because no gold standard is available; therefore, at this stage, they should be considered discriminative and not yet evaluative OHRQoL measures. However, they differ mainly in their aims and theoretical frameworks. The Child-OIDP index was developed on a large population-based sample with the aim of being used for dental health service planning. Its theoretical framework is the same as for the original OIDP, namely oral health consequences are categorised into different levels and the index measures only oral impacts on daily performances at the ultimate level [8,9]. The Child-OIDP has the advantage over the CPQ11-14 in that it specifies the different clinical causes of each oral impact and therefore the treatments needed. Although the objective of current study is to assess oral impacts of children, a broader aim of the project was to assess the implications of using measures of oral impacts to estimate dental needs of children. Therefore the Child-OIDP was selected for this study as it is specifically designed to be incorporated into a needs system.

The procedure for using the Child-OIDP began with a self-administered questionnaire carried out with all children as a group in their classroom. The questionnaire contains a list of all oral problems that children are likely to perceive and also include an open answer for any unexpected perceived problem. It was developed during a pilot study, as a modification from the one used in the original OIDP. Children were asked to identify oral problems that they perceived in the last three months. This step aimed to focus children's attention to their oral health problems and to lead to the oral impacts assessment later. Their answers here were used only as a guide to investigate oral impacts on daily performances in the next step and were referred to when they were asked about the causes of oral impacts in individual interviews. Thereafter, children were individually interviewed, irrespective of their answers at the first step, to assess oral impacts on daily life in relation to 8 daily performances. The 8 performances were: a) eating, b) speaking, c) cleaning teeth, d) relaxing, including sleeping, e) smiling, laughing and showing teeth without embarrassment, f) maintaining emotional state, g) study, including going to school and doing homework and h) contact with other people. The individual interviews were aided by 16 pictures (negative and positive pictures for each performance). If children reported an impact on any performance, the frequency of the impact and the severity of its effect on their daily life were scored. Children were also asked to identify oral problems that in their opinion, caused the impact. The oral problems were identified from the list complied in the first step of the assessment.

The oral impact score of each performance is obtained by multiplying severity and frequency scores, 0, 1, 2 or 3 each, in relation to that performance. Therefore scores can range from 0 to 9 per performance. The overall impacts score is the sum of all 8 performances (ranging from 0 to 72) divided by 72 and multiplied by 100. An alternative method of reporting the severity of oral impacts, from the same data set, is to use the 'intensity' and 'extent' of impacts. The intensity refers to the most severe impacts on any of the 8 performances or the highest performance score. It is classified into 6 levels; none, very little, little, moderate, severe and very severe (Table 1). The idea behind this is to differentiate between for example, a child with minor impacts (score of 1) on 6 performances and another child with severe impacts (score of 6) on only 1 performance. In the former case, the child will be in the 'very little', and in the latter one, in the 'severe' category. The extent refers to the number of performances with impacts (PWI) affecting a child's quality of life over the past three months. It ranges from 0 to 8 PWI. The relationships between the impact score and intensity as well as between score and extent were statistically significant (p < 0.001) [18]. Intensity and extent of impacts represent an alternative method of describing or comparing oral impacts on children. They are more straightforward and could give a simpler and clearer picture of impacts than using a single score. Therefore, they provide a more practical aspect to the OHRQoL assessment making it more easily applicable to dental service planning.

**Table 1:** Classification of the intensity of oral impacts on a performance

**The intensity of impacts**	**Severity score**		**Frequency score**	**Performance score**
Very severe	Severe (3)	×	Severe (3)	9
Severe	Severe (3)	×	Moderate (2)	6
	Moderate (2)		Severe (3)	
Moderate	Moderate (2)	×	Moderate (2)	4
	Severe (3)	×	Little (1)	3
	Little (1)		Severe (3)	
Little	Moderate (2)	×	Little (1)	2
	Little (1)		Moderate (2)	
Very little	Little (1)	×	Little (1)	1
No impact	None (0)	×	None (0)	0

## Results

1101 of the 1126 children returned positive consent forms approved by their parents. 1034 children (91.8% of the total) completed all stages of the survey. 52.4% were male and 47.6% were female. Their mean age was 11.3 years (sd = 0.6). The highest percentage of their fathers were agricultural workers or labourers (34.5%), 30.5% worked in business/private, 27.5% in governmental sectors, 2.1% did not work and 5.4% of children did not have a male guardian. The highest percentage of mothers worked in business/private (38.6%), 24.5% in agriculture, 21.1% in governmental organisations. 14.9% did not work and 1.0% did not have a female guardian.

This population had a low level of dental caries: 43.1% were caries free and the DMFT scores ranged from 0 to 12 with a median score of 1.0 and a mean of 1.5 (±1.8). Almost all children (97.0%) had a Community Periodontal Index (CPI) score of 1 or more; 84.2% had calculus. In terms of oral hygiene status, 5.4% had good, 69.1% had moderate and 25.5% had poor oral hygiene. OHI-S scores ranged from 0.5–5.5 with a median of 2.5 and mean score of 2.5 (±0.9), indicating a moderate level of oral hygiene.

The prevalence of oral impacts was high; 89.8% of children had experienced some kind of oral impact on their daily life during the past three months. There was no difference between the prevalence of impacts in girls and boys (Chi-square test). Impacts on Eating were the most prevalent (72.9%). The prevalence of impacts on Emotion (58.1%), Cleaning teeth (48.5%) and Smiling (40.1%) were also relatively high. The remaining prevalences of impacts were lower, namely Study (15.4%), Relaxing (14.7%), Contact with people (12.2%) and Speaking (9.9%) (Table 2).

**Table 2:** Prevalence, intensity and score of oral impacts in Thai school children

		**Performances**							
		
**Oral impacts on daily performances**	**Overall impacts**	Eating	Speaking	Cleaning teeth	Relaxing	Emotion	Smiling	Study	Contact
**Prevalence (%)**	89.8	72.9	9.9	48.5	14.7	58.1	40.1	15.4	12.2
**Impact intensity (% of children with impacts)**									
- Very little	15.9	27.9	37.4	33.2	37.4	43.7	25.5	57.8	49.2
- Little	31.1	39.0	33.3	38.8	44.2	37.2	28.2	31.2	38.5
- Moderate	31.7	21.8	19.2	20.8	14.3	13.9	27.4	9.7	10.7
- Severe	18.7	10.8	9.1	6.6	3.4	4.7	15.7	1.3	1.6
- Very severe	2.6	5.5	1.0	0.6	0.7	0.5	3.2	0.0	0.0
**Impact score**									
- Range	0–59.7	0–9	0–9	0–9	0–9	0–9	0–9	0–6	0–6
- Mean (sd)	8.85 (7.4)	1.87 (1.8)	0.23 (0.9)	1.13 (1.6)	0.30 (0.7)	1.17 (1.4)	1.21 (2.0)	0.25 (0.7)	0.21 (0.7)
- Percentiles (25,50,75)	2.8,7.6,12.5	0,2,2	0,0,2	0,0,0	0,0,0	0,1,2	0,0,2	0,0,0	0,0,0

### Extent and Severity of impacts

Among the children with impacts, the extent of impacts varied from 1 to 8 performances with impacts (PWI); 16.2% had 1 PWI, 23.3% had 2, 26.9% had 3 and 18.4% had 4 PWIs. Few children had 5 or more PWIs. About 1 in 5 children had severe or very severe intensity of impacts; 18.7% had severe and 2.6% had very severe intensity of impacts.15.9% had very little, 31.1% had little and 31.7% had moderate intensity of impacts (Table 2). The intensity of impacts on each performance showed that Eating and Smiling were the most severely affected while Study and Contact were the least. 16.3% of children with impacts on Eating and 18.9% of those on Smiling had severe or very severe impacts, while the same intensity was reported by 1.3–10.1% of children having impacts on other performances. 57.8% of children with impacts on Study and 49.2% of those on Contact had a very little or little level of impact intensity, whereas none had a very severe intensity of impacts on those two performances.

The distribution of overall impact scores was skewed (Table 2). They ranged from 0.0 to 59.7 with a median of 7.6 and a mean score of 8.8 (sd = 7.4). No difference in overall impact scores were identified between different sexes (Mann-Whitney U test). Mean scores of impacts on each of the 8 performances ranged from 0.21 to 1.87 (maximum possible score is 9). Mean impact score for Eating (1.87) and Smiling (1.21) were the highest while those for Study (0.25) and Contact (0.21) were the lowest (Table 2).

### 'Causes' of the impacts

There were various oral and dental problems that children perceived as the causes of their overall oral impacts (Table 3). The more prevalent problems leading to impacts were a sensitive tooth (27.9%), oral ulcers (25.8%), toothache (25.1%) and an exfoliating primary tooth (23.4%). Furthermore, oral conditions that related to appearance frequently affected children; position of teeth (20.0%) and colour of teeth (16.2%) were quite frequently cited. In addition, swollen or inflamed gums were related to overall impacts in 13.8% of children.

**Table 3:** Frequency of oral conditions perceived as causing overall oral impacts

**Oral conditions causing overall impacts**	**Frequency (%)**
Toothache (t-ache)	25.1
Sensitive tooth (t-sensitive)	27.9
Tooth decay, hole in tooth	5.0
Fractured permanent tooth	4.6
Colour of teeth (colour)	16.2
Shape or size of teeth	2.7
Position of teeth (position)	20.0
Bleeding gum (bleed)	7.4
Swollen or inflamed gum (swollen)	13.8
Calculus	0.9
Bad breath	7.2
Oral ulcer (ulcer)	25.8
Exfoliating primary tooth (exfoliat)	23.4
Tooth space (due to unerupted permanent tooth) (space)	5.3
Erupting permanent tooth	4.9
Deformity of mouth or face	0.4
Missing permanent tooth	0.7

**Figure 1:**
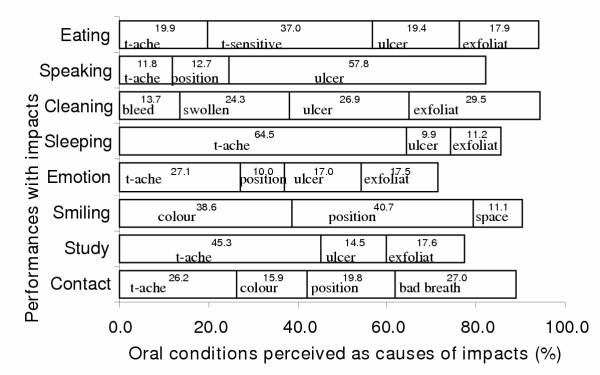
**Main oral conditions causing impacts on each of the eight performances. **Abbreviations refer to Table 3.

The main perceived causes of impacts on each of the 8 performances are shown in Figure 1. Toothache and oral ulcers were among the main perceived causes of impacts on 6 performances. The majority of impacts on Eating were caused by toothache (64.5%) and on Speaking by oral ulcers (57.8%). An exfoliating primary tooth was one of the main perceived causes of impacts on the following 5 performances; Eating (17.9%), Cleaning (29.5%), Relaxing (11.2%), Emotion (17.5%) and Study (17.6%). Position of teeth was among the main perceived causes of impacts on 3 performances; Smiling (40.7%), Contact (19.8%) and Emotion (10.0%). Space due to a non-erupted permanent tooth (after exfoliation) was one of the main reasons for impacts on Smiling (11.1%). Bad breath was the most frequent perceived cause of impacts on social Contact (27.0%).

## Discussion

The prevalence of oral impacts experienced during the past three months by the study population was very high (89.8%). This is surprising in that this was a low caries population in an area with a free accessible school dental service. Although there is no study using OHRQoL index with a population-based sample of 12 year olds to compare with, findings of previous OHRQoL studies suggest that oral impacts are very common in children of this age. In Brazilian adolescent populations, the prevalence of impacts was 32% [19,20] and 62% in Uganda [21]. In child populations, a 88% prevalence of dental pain was reported in South African 8–10-year-old school children [11] and 73% of New Zealand children with good oral status had at least one dental symptom in the past year [12]. That was higher than the 60.1% reported in Malaysian children who also had good oral status and received successful school dental services [10]. A study using the CPQ11-14 index with paedodontic patients found that all the children had oral impacts in the past three months [17]. These findings indicate that oral impacts may be higher in children than in adults. For example, compared to studies using the original OIDP index [8] with other older age groups, the prevalence of oral impacts in a Thai adult population was 73.6% [22] and 52.8% for a Thai elderly population [23]. In a UK national survey of elderly people the prevalence of OIDP impacts was 17% for edentate and 14% for dentate participants [24].

Despite the fact that oral impacts were prevalent in this Thai child population, they were not severe. For example, half of this population had Child-OIDP score less than 7.6 and half of those with impacts had very little or little intensity of impacts (Table 2). Moreover, many clinical causes that contributed to the prevalent impacts do not last long; that is oral ulcers, exfoliating teeth and spaces due to a non-erupted permanent tooth.

This study found that eating was the most important aspect of OHRQoL of children. Difficulty with eating due to oral problems was the most common impact (72.9%), and led to more severe oral impacts on children's quality of life than impacts on other performances. Oral ulcers and exfoliating primary teeth contributed to eating difficulties in nearly half of those with impacts. The finding that eating was the most common performance affected is similar to all studies using the OIDP in all age groups [19,21-24]. They are also similar to a study using the CPQ11-14 with paedodontic patients where impacts on functional limitations were more common than impacts on emotional and social well-being [17].

Difficulty with smiling was another important aspect of children's OHRQoL. It affected 40% of children. The most prevalent cause was position of teeth. Dissatisfaction with position of teeth, moreover, accounted for oral impacts in 1 in 5 of all children (Table 3). Although there is no study documenting the extent of pre-adolescent children's concern about their oral appearance, it is evident that the concern is important when they reach adolescence [25]. For example, de Oliveira and Sheiham found that adolescents with untreated malocclusions were significantly more likely to report oral impacts on their daily lives than those who had completed orthodontic treatment [26]. Chen and Hunter found that psychological impacts of oral health, such as avoiding laughing and being teased about teeth, were more prevalent in children than in adults and elderly [12].

Gum problems were the other important oral conditions affecting children's OHRQoL. More than one fifth of children perceived that bleeding and swollen gums caused oral impacts on their life, particularly in relation to difficulty cleaning, a problem experienced by nearly half of all children (Table 3, Figure 1). Children with difficulty cleaning their teeth because of gum inflammation are unlikely to achieve good levels of oral hygiene because brushing may lead to bleeding, and their gum problems would undoubtedly remain or even get worse. This problem would not be solved by the traditional dental treatment without understanding the affects of oral impacts on behaviour.

An interesting finding was that impacts relating to social dimensions, such as study being affected and contact with people, were less common and least severe. Schor suggested that children's social performances rely more on their physical and psychological performances than adults [27].

It is apparent that an important reason for the high prevalence of oral impacts in children is natural processes such as exfoliating primary teeth or spaces due to a non-erupted permanent tooth. They contributed largely to the high incidence of impacts in these pre-adolescent children. On the other hand, these conditions were not reported as important causes of oral impacts in other age groups [19,22]. The findings on the other clinical causes of oral impacts in this study was consistent with what Jaafar found in Malaysian children, namely, toothache and oral ulcers [10]. Moreover, it is noteworthy that despite the fact that this was a low caries population having access to free dental service, sensitive teeth and toothache were frequently reported causes across the various impacts, particularly so with respect to the more common impact of difficulties with eating.

Although children could often not specify precisely which impairments led to impacts, the question of perceived clinical causes should exclude impacts from some conditions which are definitely not related to actual impairments as well as to treatment needs. For example, toothache, ulcers and conditions relating to appearance definitely require different treatment and could be easily differentiated. However, the accuracy of detecting perceived impairment is limited in a population-based study, while it can be improved at the individual level of investigation.

The specific age group under investigation, particularly in relation to their stage of development, may have influenced the high prevalence of oral impacts. Developmental changes unavoidably affect HRQoL between childhood and adolescence [28]. Maturity and an increase in age generate a more sophisticated understanding and perceptions about health and illness [29]. Therefore, perceptions about health and quality of life of children change as they mature [28,30]. This might make younger children more sensitive to oral symptoms than older age groups. Because of those considerations the modification of the Child-OIDP addressed the main possible problems that might arise when employing adult measures with children [30,31]. They include the adjustment of the 8 items of daily performances, simplification of rating scales, decrease of the time frame and rearrangement and clarification of the complex questions that were beyond the capability of children under 12 years according to Piaget's cognitive development theory [32]. Moreover, the use of pictures as aids is considered of value when interviewing children [33,34]. In addition to the modification, another advantage of the Child-OIDP lies in its conceptual framework where oral health consequences are divided into three levels; the first level represents oral problems (such as tooth decay), the second or intermediate level represents symptoms (such as pain) and the third or "ultimate level" represents difficulty in daily performances. The index measures impact at the ultimate level only, which could reduce double scoring, by not measuring twice the same impacts experienced at different levels. For example, pain is not scored whereas difficulty with eating due to pain is scored. In addition, this approach could reduce the uncertainty of children's perception and interpretation and therefore make the index more applicable for children [35]. Fink explained that HRQoL can be measured through different types of information. Measuring impacts on daily functioning is more objective and reliable than measuring reported health problems or symptoms which are more influenced by individuals' perception and interpretation [36]. Thus, HRQoL measures for children that involve subjective reported problems or symptoms such as pain are frequently problematic, because children's interpretation and perception about health differ from adults [30]. On the other hand, HRQoL measures that focus on information about functioning, such as the Sickness Impact Profile, may readily be applied to children as well as adults [37]. Therefore, to reduce a problem with children's interpretation about their health or symptoms, the technique of assessing HRQoL based on activities of daily living is appropriate [35].

## Conclusions

The prevalence of oral impacts on daily performances in this child population was very high. Oral impacts affected children's quality of life mainly through difficulty eating and smiling. There are various oral conditions that contributed significantly to the incidence of impacts, namely, sensitive teeth, toothache, oral ulcers and exfoliating primary teeth. Although the prevalence of impacts was high, the severity was not; many children had their quality of life affected at low levels. This reveals a need for further longitudinal studies to better understand and interpret OHRQoL measures in children.

## Authors' contributions

SG carried out all work including data collection, data analysis and writing the paper. GT supervised the project and assisted writing. AS initiated the idea of, and supervised the project and edited writing.
